# Supplementary material for: Content-rich biological network constructed by mining PubMed abstracts
Source: BMC Bioinformatics. 2004 Oct 8;5:147. doi: 10.1186/1471-2105-5-147 (PMC528731; doi:10.1186/1471-2105-5-147)
Supplement: Additional File 2 — The original results of the above study (non-essential files are deleted to keep the file size under the limit set by BMC bioinformatics). [file 1471-2105-5-147-S2.bz2 › chilibotAdditionalFile2/dip10/111ID8645696E455/html/ATPA_ATPD.html]

 


 **ATPA** and **ATPD** 
  
Found 7 abstracts in PubMed, retrieved 7.  
 

 What does Google say? 
 PDF only 
| .edu only 

---

**Interactive relationship** (e.g. stimulation, inhibition, etc)

**Non-interactive relationship** (e.g. studied together, co-existance, homology, etc.)

- These fragments contain six closely linked reading frames that were identified as atpI, atpH, atpG, atpF,  **atpD** , and  **atpA** , coding for subunits IV, III, II, I, delta, and alpha, respectively.  Ref: 1532839 J Mol Biol, 1992
- Subsequently, a PCR product from  **atpA**  to  **atpD**  was identified.  Ref: 11016603 Int J Food Microbiol, 2000
- The C. thermoaceticum atp operon contained nine genes arranged in the order atpI i, atpB a, atpE c, atpF b, atpH delta,  **atpA**  alpha, atpG gamma,  **atpD**  beta, and atpC epsilon.  Ref: 9171425 J Bacteriol, 1997
- Eight genes atpI, atpB, atpE 1, atpE 2, atpE 3, atpF, atpH, and  **atpA**  upstream of and contiguous with the previously described genes atpG,  **atpD** , and atpC were cloned from chromosomal DNA of Acetobacterium woodii.  Ref: 10567365 J Biol Chem, 1999
